# Cognitive Frailty Predicts Incident Dementia among Community-Dwelling Older People

**DOI:** 10.3390/jcm7090250

**Published:** 2018-08-30

**Authors:** Hiroyuki Shimada, Takehiko Doi, Sangyoon Lee, Hyuma Makizako, Liang-Kung Chen, Hidenori Arai

**Affiliations:** 1 Department of Preventive Gerontology, Center for Gerontology and Social Science, National Center for Geriatrics and Gerontology, Obu 474-8511, Japan; take-d@ncgg.go.jp (T.D.); sylee@ncgg.go.jp (S.L.); 2 School of Health Sciences, Faculty of Medicine, Kagoshima University, Kagoshima 890-8544, Japan; makizako@health.nop.kagoshima-u.ac.jp; 3 Center for Geriatrics and Gerontology, Taipei Veterans General Hospital, Taipei City 112, Taiwan; lkchen2@vghtpe.gov.tw; 4 National Center for Geriatrics and Gerontology, Obu 474-8511, Japan; harai@ncgg.go.jp

**Keywords:** dementia, cognitive frailty, elderly, functional assessment, epidemiology

## Abstract

Cognitive frailty, defined as the presence of both frailty and cognitive impairment, is a risk factor for adverse events in older adults. However, prevalence rates of cognitive frailty are low (1.1–2.5%), so primary screening is unsuitable in community settings. The aim of the study was to examine whether a new definition of cognitive frailty, which was developed for primary screening, is useful to predict incident dementia in community-dwelling older adults. A total of 4570 older adults participated in the study (2326 women; average age, 71.9 ± 5.5 years). We defined physical frailty as the presence of ≥1 of the following symptoms: slow walking speed and muscle weakness. Cognitive impairment was defined as ≥1 symptom of cognitive impairment, indicated by an age- and education-adjusted score that was ≥1.5 standard deviations below the reference threshold in word list memory, attention, executive function, and processing speed tests. Cognitive frailty was defined as comorbid physical frailty and cognitive impairment. The incidence of dementia was determined using data collected by the Japanese Health Insurance System over 36 months. The prevalence rates of physical frailty, cognitive impairment, and cognitive frailty were 17.5%, 15.3%, and 9.8%, respectively. Cognitive impairment (hazard ratio [HR]: 2.06, 95% confidence interval [95% CI]: 1.41–3.02) and cognitive frailty (HR: 3.43, 95% CI: 2.37–4.97) were found to be significant risk factors for dementia. However, the association between dementia and physical frailty was not significant (HR: 1.13, 95% CI: 0.76–1.69). Individuals with comorbid physical frailty and cognitive impairment could have a higher risk of dementia than healthy older adults or older adults with either physical frailty or cognitive impairment alone.

## 1. Introduction

Cognitive decline is associated with physical frailty in older adults [1], and cognitive impairment and physical frailty are often comorbid conditions in older people [2,3]. The first definition of cognitive frailty as a condition affecting older adults was provided by the International Consensus Group on Cognitive Frailty, which proposed identification of cognitive frailty as a clinical symptom characterized by the comorbidity of physical frailty and cognitive impairment [4]. The International Association of Gerontology and Geriatrics consensus conference further defined cognitive frailty as reduced cognitive function due to physical or brain disease, or accelerated brain aging in the absence of evident brain disease [5]. In older adults, a series of different subclinical and age-related comorbidities may exacerbate multisystem physiological decline, resulting in homeostatic imbalance [6]. This multidimensional physiological decline, which reflects a nonspecific state of vulnerability, may increase the risk of not only physical frailty but also cognitive impairment.

Several previous studies have investigated the prevalence of cognitive frailty and the association of cognitive frailty with activities of daily living (ADL) [7] or the incidence of dementia [8] in older adults. Previously, we reported that the overall prevalence rate of cognitive frailty was only 1.1% in a cohort of 4072 older adults [8], and other population-based studies estimated the prevalence of cognitive frailty to be 1.8–2.5% [9,10]. We considered the criteria of cognitive frailty with low prevalence rates, which increased the risk of false-negative results, to be unsuitable for primary screening in the community. 

Many studies use the Fried/Cardiovascular Health Study criteria to define physical frailty, which includes slow gait velocity, low physical activity, weakness, shrinking/weight loss, and exhaustion [11]. In the studies that used the Fried criteria, the presence of cognitive impairment with frailty and prefrailty was associated with increased risk of incident mild cognitive impairment (MCI) or dementia [12] and disability [9], although prefrailty without cognitive impairment was not associated with increased risk of these adverse events. Furthermore, slow gait and cognitive impairment combined showed a higher risk of incident dementia compared with frailty and cognitive frailty [13], because slow gait as an individual component of the physical frailty phenotype has been associated with incident cognitive impairment [14] and non-Alzheimer’s disease (AD) dementia [15]. Although the questionnaire is relatively quick to administer, a performance-based assessment could determine actual physical capacity and might more accurately predict subsequent disability in community-dwelling older people [16]. Guralnik et al. reported that measures of physical performance may help identify older persons with a preclinical stage of disability who may benefit from interventions to prevent the development of frank disability [17]. 

Based on these findings, we developed a new operational definition of cognitive frailty as follows: physical frailty; presence of slow walking speed or muscle weakness; cognitive impairment [18]; and signs of impairment in word list memory, attention, executive function, or processing speed in the National Center for Geriatrics and Gerontology-Functional Assessment Tool (NCGG-FAT) [19,20]. This new definition has less stringent criteria than those we previously reported in order to improve its suitability as a primary screening tool [8]. In the present study, we aimed to examine whether a new definition of cognitive frailty, which was developed for primary screening, is useful to predict incident dementia in the community-dwelling older adults, using data from a Japanese national cohort study, the National Center for Geriatrics, and Gerontology-Study of Geriatric Syndromes (NCGG-SGS) [21]. We hypothesized that individuals with cognitive frailty would have higher incidence rates of dementia than healthy older adults or older adults with physical frailty or cognitive impairment alone.

## 2. Experimental Section

### 2.1. Participants

The study population comprised 4570 community-dwelling older adults aged ≥65 years old recruited from Obu, Japan, for the NCGG-SGS [21]. The inclusion criteria were residence in Obu and age ≥65 years at the time of examination (August 2011–February 2012, June 2013). Exclusion criteria included a history of AD (*n* = 140) or Parkinson’s disease (*n* = 18); a Mini–Mental State Examination (MMSE) [22] score <21 (*n* = 146), which might reflect moderate dementia [23]; and responses with missing data for the cognitive frailty assessment and other measurements (*n* = 230). Thus, 534 of the initial 5104 participants were excluded, and data from 4570 older adults were included in the final analysis (2326 women; average age ± standard deviation, 71.9 ± 5.5 years, range 65–97 years). 

### 2.2. Operational Definition of Cognitive Frailty

Participants were first divided into two groups: a physical frailty group, comprising participants with slow walking speed or muscle weakness, and a non-physical frailty group, comprising participants with neither slow walking speed nor muscle weakness. Walking speed was measured using a stopwatch. The average of five walking speed times was used as a representative value, with slowness defined according to a cutoff of <1.0 m·s^−1^ [3,24]. The European Working Group on Sarcopenia in Older People and Asian Working Group for Sarcopenia-algorithms uses a cut-off gait speed of 0.8 m·s^−1^ to diagnose sarcopenia in older adults. However, the number of people with a gait speed of <0.8 m·s^−1^ (4.3%) in our sample of community-dwelling older adults was lower than that of people with a gait speed of >1.0 m·s^−1^ (15.8%). The cut-off walking speed was 1 m·s^−1^, which is the critical point for the prediction of future functional decline in community-dwelling older people according to previous studies [24,25,26,27,28,29]. The International Working Group on Sarcopenia criteria [30] and the Sarcopenia, Cachexia, and Wasting Disorders definition [31] set the cut-off walking speed at 1.0 m·s^−1^ for the diagnosis of sarcopenia. For the above reasons, the cut-off walking speed was set to 1.0 m·s^−1^ in this study. Weakness was defined according to maximum handgrip strength (kg), determined using a Smedley-type handheld dynamometer (GRIP-D; Takei Scientific Instruments Co., Ltd., Niigata, Japan). Sex-specific cutoff values for maximum handgrip strength to establish weakness were <26 kg for men and <18 kg for women [32].

Next, we performed cognitive screening using the NCGG-FAT, which comprises multidimensional cognitive tasks to assess (1) memory: immediate and delayed word list memory; (2) attention: trail making test-part A; (3) executive function: trail making test-part B; and (4) processing speed: digit symbol substitution test. NCGG-FAT has been shown to have high test–retest reliability [19], moderate-to-high criterion-related validity [19], and predictive validity for dementia [20] in older adults. Participants were given approximately 20 min to complete the tests. Prior to study commencement, the authors trained all staff to correctly administer the assessment measures, with study assistants assessing participants’ cognitive functioning in community facilities such as community halls. For all tests conducted in this study, we used established standardized thresholds to define impairment in the corresponding domain in a population-based cohort comprising community-dwelling older adults (score >1.5 standard deviations [SD] below the age- and education-specific means) [3]. Participants without deficits were considered cognitively intact, whereas those with one deficit were considered to have cognitive impairment. We defined cognitive frailty as the coexistence of physical frailty and cognitive impairment [18]. Participants were categorized into the following four final groups: healthy group, physical frailty group, cognitive impairment group, and cognitive frailty group.

### 2.3. Measurement of the Incidence of Dementia

In Japan, all adults aged ≥65 years have one of the following types of public health insurance: health insurance for employed individuals (Employees’ Health Insurance), national health insurance for unemployed and self-employed individuals 65–74 years old (Japanese National Health Insurance), or health care for individuals ≥75 years old (Later-Stage Medical Care) [33]. The Japanese National Health Insurance and Later-Stage Medical Care systems were checked on a monthly basis for newly reported cases of incident dementia (AD or other dementia subtypes). Participants were considered to have dementia on the basis of a diagnosis by medical doctors according to the International Classification of Diseases-10. Participants without dementia at baseline and those who were diagnosed with dementia over a 36-month follow-up period according to Japanese Health Insurance System data were considered to have incident dementia.

### 2.4. Potential Confounding Factors

Dementia may be due to several factors occurring together over an extended period of time. Demographic variables, chronic medical conditions, lifestyle, psychosocial factors, and functional limitations are associated with the incidence of dementia in older adults [5,34]. The multivariate models for examining dementia in the present study included the following covariates: older age; sex; education level; chronic medical illnesses; medications; living alone; currently smoking; no or infrequent exercise; not being engaged in paid work; subjective memory complaints; depressive symptoms; enrolled in the Japanese-certified, public, long-term care insurance system and requiring support or care; and the inability to perform basic daily living tasks such as eating, grooming, bathing, walking, and climbing stairs. The following self-reported chronic medical illnesses were also entered into the following models: heart disease, pulmonary disease, hypertension, hyperlipidemia, diabetes mellitus, osteoarthritis, stroke, and depression. The Geriatric Depression Scale-15 (GDS) was used to assess depressive symptoms [35].

### 2.5. Statistical Analysis

We used analysis of variance, Student’s *t* test, and the χ^2^ test to detect significant differences in baseline characteristics according to frailty status and differences between participants with and without incident dementia. Participants who moved away or died during the follow-up period were excluded from univariate analyses for comparison of dementia status (n = 4401). The *χ*^2^ test with adjusted standardized residuals was used to determine whether cognitive frailty significantly affected the incidence of dementia. Residuals followed the *t* distribution, with *t* > 1.96 accepted as indicating *p* < 0.05 and *t* > 2.56 accepted as indicating *p* < 0.01.

We calculated the cumulative dementia incidence rate during the follow-up period according to participants’ baseline frailty status. Intergroup differences were estimated using the log-rank test. To analyze associations between cognitive frailty and incidence of dementia, we used Cox proportional-hazards regression models. We also used a multiple adjustment model that was adjusted for demographic variables, primary diseases, lifestyle, and psychological variables as possible confounding factors. Adjusted hazard ratios (HRs) for dementia incidence and their 95% confidence intervals (95% CI) were estimated. All analyses were performed using IBM SPSS version 24.0 (IBM Japan, Tokyo, Japan). In all analyses, *p* < 0.05 was considered to indicate statistical significance.

## 3. Results

**Figure 1:**
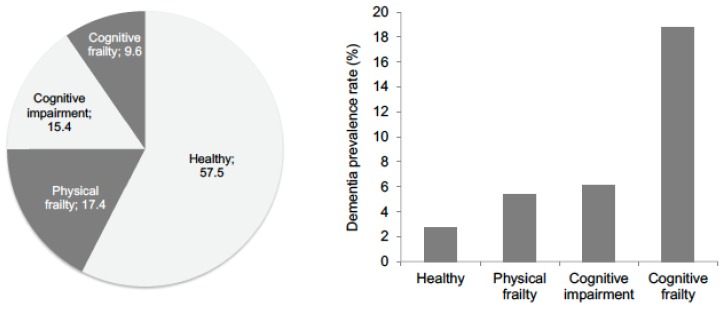
Prevalence of cognitive frailty and dementia according to frailty status.

The healthy, physical frailty, cognitive impairment, and cognitive frailty groups accounted for 2630 (57.5%), 796 (17.4%), 703 (15.4%), and 441 (9.6%) of the total study population, respectively (Figure 1). Dementia was diagnosed in 241 participants (5.3%); 42 participants (0.9%) moved away from Obu, and 127 participants (2.8%) without dementia died during the follow-up period. The healthy, physical frailty, cognitive impairment, and cognitive frailty groups had dementia prevalence rates of 2.7%, 5.4%, 6.1%, and 18.8%, respectively (Figure 1). Residual analyses indicated that, among all four groups, the cognitive frailty group had the higher proportion of participants with incident dementia (all *p* < 0.01), while the healthy group had the lowest proportion of participants with incident dementia (all *p* < 0.01).

Possible confounding factors for dementia in participants grouped according to frailty status are shown in Table 1. Significant differences were observed among the four groups (i.e., the healthy, physical frailty, cognitive impairment, and cognitive frailty groups) with regard to age, sex, education level, heart disease, hypertension, diabetes, osteoarthritis, stroke, medications, living alone, currently smoking, exercise habit, engaging in paid work, subjective memory complaints, GDS score, certified long-term care insurance, and functional limitation. Significant differences were also noted between participants with and without incident dementia for age, sex, education level, heart disease, pulmonary disease, hypertension, stroke, depression, medications, living alone, exercise habit, engaging in paid work, subjective memory complaints, GDS score, and certified long-term care insurance (Table 2).

**Figure 2:**
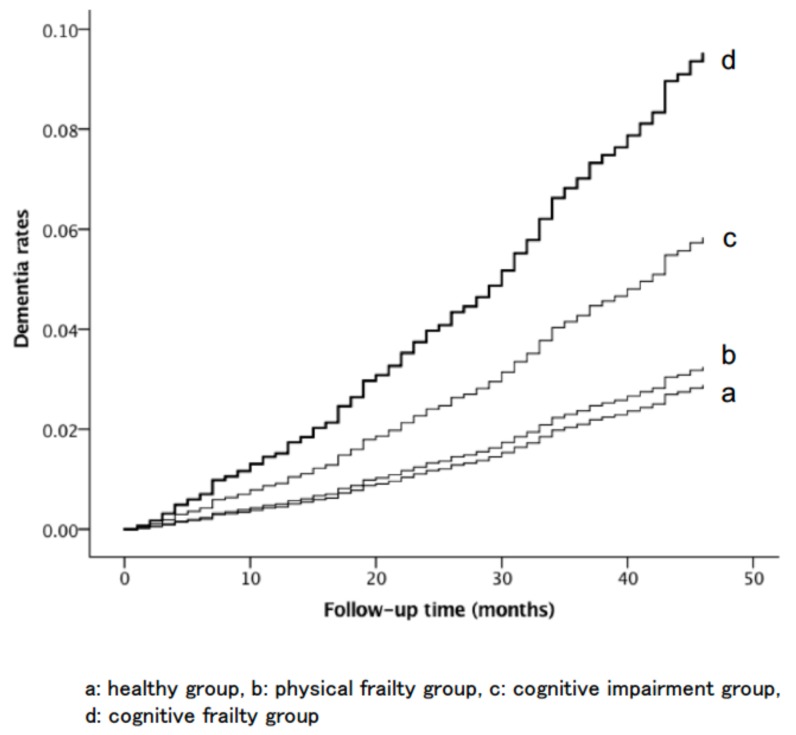
Cox proportional hazard estimates for the incidence rates of dementia according to frailty status.

The dementia survival rates according to the four groups are shown in Figure 2. Kaplan–Meier analysis and log-rank tests revealed that the proportion of patients with dementia was significantly higher in the physical frailty, cognitive impairment, and cognitive frailty groups than in the healthy group (*p* < 0.001). The incidence rate of dementia was also significantly different between the cognitive frailty and physical frailty groups (*p* < 0.001) and between the cognitive frailty and cognitive impairment groups (*p* < 0.001). However, no significant difference in the incidence of dementia was observed between the physical frailty and cognitive impairment groups (*p* = 0.602).

The associations between frailty and the incidence of dementia were analyzed using Cox proportional-hazards regression models (Table 3). In the fully adjusted model, HRs and 95%CIs were 1.13 and 0.76–1.69, 2.06 and 1.41–3.02, and 3.43 and 2.37–4.97 for the physical frailty, cognitive impairment, and cognitive frailty groups, respectively, compared with healthy participants. No significant association was found between physical frailty and dementia incidence. Older age, female sex, history of stroke, subjective memory complaints, and higher GDS score were positively correlated with the incidence of dementia in older adults.

## 4. Discussion

In this prospective study, dementia risk was significantly associated with cognitive impairment and cognitive frailty, and the participants with cognitive frailty had a higher HR than those with cognitive impairment. Several studies in patients with MCI investigated the relationship between cognitive impairment and incident dementia, revealing that measures of episodic memory [36,37], semantic memory [37,38], and executive function [39,40] predict conversion to dementia. These results suggest that multi-domain cognitive tests, such as those examining general cognitive function, memory, and executive function, are useful for assessing dementia risk in older individuals. Many computer-based test batteries have been either developed or already used to screen for cognitive decline in elderly individuals [41]. The NCGG-FAT, administered as an application that runs on an electronic tablet, consists of memory, attention, executive function, and processing speed domains. All of the NCGG-FAT tests have well-established standardized thresholds to define cognitive impairment in the corresponding domain for population-based cohorts consisting of community-dwelling older adults. Validated test batteries such as the frailty criteria or NCGG-FAT, used for early detection of the risk for physical and cognitive decline, could help identify and possibly delay the onset of dementia in both community and clinical settings.

In the present study, the association of dementia risk with both cognitive impairment and cognitive frailty remained significant after adjusting for age, sex, education level, depressive mood, and chronic medical illnesses. The dementia incidence risk in the cognitive impairment and cognitive frailty groups was 2.1 and 3.4 times as high as that in the healthy group, respectively. The results of the multivariate analyses showed clearly that individuals with comorbidity of physical frailty and cognitive impairment have a higher risk of dementia than healthy older adults or older adults with either physical frailty or cognitive impairment alone. The observations presented here are consistent with those of previous prospective studies indicating that older adults with motoric cognitive risk syndrome, a disorder with a similar definition and treatment to cognitive frailty, are at higher risk for incident dementia, especially incident vascular dementia [42]. 

Neuropathologically, AD is known to include brain regions involved in motor control, such as the substantia nigra, primary and supplementary motor cortices, and striatum. Furthermore, diffuse striatal plaques have been shown to occur relatively early in the progression of AD pathology [43]. A clinicopathological study indicated that physical frailty proximate to death was correlated with the level of AD pathology on postmortem examination, and the association was similar in persons with and without dementia. These findings raise the possibility that AD pathology may contribute to frailty, or that frailty and AD pathology share a common etiopathogenesis [44]. For instance, previous studies have indicated that risk factors for cardiovascular disease and common vascular diseases, which are prevalent and can lead to physical and cognitive decline, are significantly correlated with frailty [45] and AD [46]. Moreover, increased levels of inflammatory markers such as C-reactive protein and proinflammatory interleukins are also common and have been implicated in frailty [47], cognitive impairment [48], and AD [49,50]. 

Control of these diseases may be important in preventing cognitive frailty in older adults. Physical frailty and cognitive decline are also associated with genetic factors, as well as environmental conditions such as nutrition, activity, and sleep [6,51]. Additionally, multifactorial and multilevel interventions addressing physical and cognitive domains, such as diet, exercise, cognitive training, and vascular risk monitoring, may be useful for identifying and possibly delaying dementia among older adults, especially among those with cognitive frailty [52,53]. Thus, health-care providers are advised to perform physical, as well as cognitive, assessments to evaluate dementia risk and to deliver health-care services targeted at high-risk individuals, especially in cognitively frail older persons. Further research is required to identify novel candidate biomarkers, which are necessary for early detection of physical frailty and cognitive impairment.

This study has several limitations. First, participants were not recruited randomly, which may have resulted in underrepresentation of individuals with physical frailty and cognitive impairment, because the participants were healthy enough to receive health checkups in their community. Second, we could not exclude individuals with dementia at baseline, except for those with AD; however, older adults with baseline MMSE score <21 were excluded from the study. Third, we did not collect data about dementia subtypes such as AD, vascular dementia, dementia with Lewy bodies, and frontotemporal dementia; therefore, inferences could not be made regarding correlations between frailty and dementia pathology. Fourth, we were unable to verify medical records and asymptomatic aberrant behavior for some participants. Fifth, information about participants’ medical conditions and comorbidities were collected via self-reports, and we were unable to confirm these data, because we did not have access to their medical records. However, a previous study showed that self-reported medical conditions were well-correlated with actual medical diagnoses [54]. Finally, according to previous systematic review [55], the prevalence of frailty based on the Fried criteria among older people ranged from 4% in a United States study to 27.3% in a Spanish study. The other systematic review identified that the prevalence of frailty in individual studies ranged 4.6% to 9.5% in Japanese cohort studies [56]. The results suggested that Asian cohorts are likely more homogeneous than European or American cohorts, accordingly, this research must be interpreted in light of these results.

This study also has several strengths. First, our findings are consistent with comprehensive geriatric assessments designed to identify frailty and cognitive impairment. To the best of our knowledge, this is the first Asian study to examine correlations between cognitive frailty and the incidence of dementia in a large, population-based sample. Our results indicate that individuals with comorbid physical frailty and cognitive impairment have a higher risk of incident dementia compared with both healthy older adults and older adults with either physical frailty or cognitive impairment alone.

## 5. Conclusions

Cognitive frailty was defined as comorbid physical frailty and cognitive impairment. We characterized physical frailty as ≥1 symptom of slow walking speed and muscle weakness. Cognitive impairment was defined as ≥1 symptom of cognitive impairment, indicated by an age- and education-adjusted score that was ≥1.5 SD below the reference threshold. The incidence of dementia and cognitive impairment was significantly correlated with that of cognitive frailty; however, the association between dementia and physical frailty was not significant. The multivariate analyses showed clearly that individuals with comorbid physical frailty and cognitive impairment have a higher risk of dementia than healthy older adults or older adults with either physical frailty or cognitive impairment alone. Our results suggest that comprehensive functional assessment, including frailty phenotype and cognition, can help identify dementia risk in older adults.

## Figures and Tables

**Table 1:** Comparisons of baseline characteristics according to frailty status.

Items	Robust (*n* = 2561)	Physical Frailty (*n* = 752)	Cognitive Impairment (*n* = 676)	Cognitive Frailty (*n* = 412)	*p* Value
Age, years *	70.6 (4.5)	74.4 (6.3)	71.0 (4.6)	75.7 (6.5)	<0.001
Sex, female **	1266 (49.4)	449 (59.7)	306 (45.3)	246 (59.7)	<0.001
Education, years *	11.7 (2.5)	11.0 (2.5)	11.3 (2.4)	10.3 (2.5)	<0.001
Heart disease, yes **	400 (15.6)	146 (19.4)	105 (15.5)	92 (22.3)	0.001
Pulmonary disease, yes **	283 (11.1)	94 (12.5)	60 (8.9)	48 (11.7)	0.170
Hypertension, yes **	1098 (42.9)	388 (51.6)	311 (46.0)	231 (56.1)	<0.001
Hyperlipidemia, yes **	1091 (42.6)	331 (44.0)	264 (39.1)	161 (39.1)	0.139
Diabetes, yes **	296 (11.6)	132 (17.6)	84 (12.4)	68 (16.5)	<0.001
Osteoarthritis, yes **	334 (13.0)	135 (18.0)	87 (12.9)	85 (20.6)	<0.001
Stroke, yes **	84 (3.3)	42 (5.6)	48 (7.1)	49 (11.9)	<0.001
Depression, yes **	67 (2.6)	22 (2.9)	18 (2.7)	14 (3.4)	0.819
Medications *	1.8 (1.9)	2.6 (2.4)	2.0 (2.0)	3.0 (2.4)	<0.001
Living alone, yes **	210 (8.2)	96 (12.8)	50 (7.4)	73 (17.7)	<0.001
Current smoking, yes **	244 (9.5)	53 (7.0)	90 (13.3)	43 (10.4)	0.001
Exercise habit, no **	1437 (56.1)	554 (73.7)	429 (63.5)	336 (81.6)	<0.001
Engaging in paid work, no **	1716 (67.0)	579 (77.0)	446 (66.0)	318 (77.2)	<0.001
Subjective memory complaints, yes **	464 (18.1)	171 (22.7)	157 (23.2)	116 (28.2)	<0.001
Geriatric depression scale-15, score *	2.4 (2.3)	3.4 (2.8)	2.8 (2.6)	4.3 (3.1)	<0.001
Japanese-certified public long-term care insurance system, yes **	6 (0.2)	36 (4.8)	3 (0.4)	35 (8.5)	<0.001
Functional limitations, yes **	4 (0.2)	4 (0.5)	1 (0.1)	5 (1.2)	0.003

* Mean (SD), ** Number (%).

**Table 2:** Comparisons between the participants with and without dementia.

Items	Participants with Dementia (*n* = 241)	Participants without Dementia (*n* = 4160)	*p* Value
Age, years *	76.4 (5.9)	71.5 (5.2)	<0.001
Sex, female **	143 (59.3)	2124 (51.1)	0.012
Education, years *	10.6 (2.6)	11.5 (2.5)	<0.001
Heart disease, yes **	57 (23.7)	686 (16.5)	0.004
Pulmonary disease, yes **	39 (16.2)	446 (10.7)	0.008
Hypertension, yes **	127 (52.7)	1901 (45.7)	0.034
Hyperlipidemia, yes **	99 (41.1)	1748 (42.0)	0.774
Diabetes, yes **	33 (13.7)	547 (13.1)	0.808
Osteoarthritis, yes **	45 (18.7)	596 (14.3)	0.063
Stroke, yes **	28 (11.6)	195 (4.7)	<0.001
Depression, yes **	13 (5.4)	108 (2.6)	0.010
Medications *	2.9 (2.5)	2.0 (2.1)	<0.001
Living alone, yes **	34 (14.1)	395 (9.5)	0.019
Current smoking, yes **	22 (9.1)	408 (9.8)	0.730
Exercise habit, no **	172 (71.4)	2584 (62.1)	0.004
Engaging in paid work, no **	194 (80.5)	2865 (68.9)	<0.001
Subjective memory complaints, yes **	92 (38.2)	816 (19.6)	<0.001
Geriatric depression scale-15, score *	4.2 (3.1)	2.7 (2.5)	<0.001
Japanese-certified public long-term care insurance system, yes **	19 (7.9)	61 (1.5)	<0.001
Functional limitations, yes **	1 (0.4)	13 (0.3)	0.784

* Mean (SD), ** Number (%).

**Table 3:** Hazard ratios of dementia for confounding factors.

Items	Hazard Ratio (95% CI)	*p*-Value
Frail vs. Healthy	1	<0.001
Physical frailty	1.13 (0.76–1.69)	0.555
Cognitive impairment	2.06 (1.41–3.02)	<0.001
Cognitive frailty	3.43 (2.37–4.97)	<0.001
Age, years *	1.10 (1.08–1.13)	<0.001
Sex, female	1.35 (1.01–1.81)	0.042
Education, years *	1.00 (0.94–1.05)	0.872
Heart disease, yes	1.03 (0.75–1.41)	0.878
Pulmonary disease, yes	1.26 (0.89–1.78)	0.201
Hypertension, yes	0.93 (0.71–1.23)	0.622
Hyperlipidemia, yes	0.93 (0.71–1.22)	0.611
Diabetes, yes	0.91 (0.62–1.33)	0.627
Osteoarthritis, yes	0.83 (0.59–1.18)	0.299
Stroke, yes	1.62 (1.07–2.45)	0.022
Depression, yes	1.58 (0.88–2.84)	0.125
Medications *	1.06 (1.00–1.13)	0.059
Living alone, yes	0.81 (0.56–1.19)	0.279
Current smoking, yes	1.26 (0.79–1.99)	0.333
Exercise habit, no	0.90 (0.67–1.21)	0.485
Engaging in paid work, no	1.05 (0.75–1.47)	0.795
Subjective memory complaints, yes	1.67 (1.25–2.22)	<0.001
Geriatric depression scale-15, score *	1.07 (1.02–1.12)	0.005
Japanese-certified public long-term care insurance system, yes	0.94 (0.56–1.58)	0.812
Functional limitations, yes	0.51 (0.07–3.78)	0.508

* Mean (SD), CI, confidence interval.

## References

[1] Auyeung T.W., Lee J.S., Kwok T., Woo J. (2011). Physical frailty predicts future cognitive decline—A four-year prospective study in 2737 cognitively normal older adults. J. Nutr. Health Aging.

[2] Malmstrom T.K., Morley J.E. (2013). The frail brain. J. Am. Med. Dir. Assoc..

[3] Shimada H., Makizako H., Doi T., Yoshida D., Tsutsumimoto K., Anan Y., Uemura K., Ito T., Lee S., Park H. (2013). Combined prevalence of frailty and mild cognitive impairment in a population of elderly Japanese people. J. Am. Med. Dir. Assoc..

[4] Kelaiditi E., Cesari M., Canevelli M., van Kan G.A., Ousset P.J., Gillette-Guyonnet S., Ritz P., Duveau F., Soto M.E., Provencher V. (2013). Cognitive frailty: Rational and definition from an (I.A.N.A./I.A.G.G.) international consensus group. J. Nutr. Health Aging.

[5] Morley J.E., Morris J.C., Berg-Weger M., Borson S., Carpenter B.D., Del Campo N., Dubois B., Fargo K., Fitten L.J., Flaherty J.H. (2015). Brain health: The importance of recognizing cognitive impairment: An IAGG consensus conference. J. Am. Med. Dir. Assoc..

[6] Clegg A., Young J., Iliffe S., Rikkert M.O., Rockwood K. (2013). Frailty in elderly people. Lancet.

[7] Shimada H., Makizako H., Lee S., Doi T., Lee S., Tsutsumimoto K., Harada K., Hotta R., Bae S., Nakakubo S. (2016). Impact of cognitive frailty on daily activities in older persons. J. Nutr. Health Aging.

[8] Shimada H., Makizako H., Tsutsumimoto K., Doi T., Lee S., Suzuki T. (2018). Cognitive frailty and incidence of dementia in older persons. J. Prev. Alzheimers Dis..

[9] Feng L., Zin Nyunt M.S., Gao Q., Feng L., Yap K.B., Ng T.P. (2017). Cognitive frailty and adverse health outcomes: Findings from the Singapore Longitudinal Ageing Studies (SLAS). J. Am. Med. Dir. Assoc..

[10] Solfrizzi V., Scafato E., Seripa D., Lozupone M., Imbimbo B.P., D’Amato A., Tortelli R., Schilardi A., Galluzzo L., Gandin C. (2017). Reversible cognitive frailty, dementia, and all-cause mortality. The Italian longitudinal study on aging. J. Am. Med. Dir. Assoc..

[11] Fried L.P., Tangen C.M., Walston J., Newman A.B., Hirsch C., Gottdiener J., Seeman T., Tracy R., Kop W.J., Burke G. (2001). Frailty in older adults: Evidence for a phenotype. J. Gerontol. Ser. A Biol. Sci. Med. Sci..

[12] Feng L., Nyunt M.S., Gao Q., Feng L., Lee T.S., Tsoi T., Chong M.S., Lim W.S., Collinson S., Yap P. (2017). Physical frailty, cognitive impairment, and the risk of neurocognitive disorder in the Singapore Longitudinal Ageing Studies. J. Gerontol. Ser. A Biol. Sci. Med. Sci..

[13] Montero-Odasso M.M., Barnes B., Speechley M., Muir Hunter S.W., Doherty T.J., Duque G., Gopaul K., Sposato L.A., Casas-Herrero A., Borrie M.J. (2016). Disentangling cognitive-frailty: Results from the gait and brain study. J. Gerontol. A Biomed. Sci. Med. Sci..

[14] Buracchio T., Dodge H.H., Howieson D., Wasserman D., Kaye J. (2010). The trajectory of gait speed preceding mild cognitive impairment. Arch. Neurol..

[15] Verghese J., Lipton R.B., Hall C.B., Kuslansky G., Katz M.J., Buschke H. (2002). Abnormality of gait as a predictor of non-Alzheimer’s dementia. N. Engl. J. Med..

[16] Shimada H., Makizako H., Doi T., Tsutsumimoto K., Suzuki T. (2015). Incidence of disability in frail older persons with or without slow walking speed. J. Am. Med. Dir. Assoc..

[17] Guralnik J.M., Ferrucci L., Simonsick E.M., Salive M.E., Wallace R.B. (1995). Lower-extremity function in persons over the age of 70 years as a predictor of subsequent disability. N. Engl. J. Med..

[18] Liu L.K., Lee W.J., Wu Y.H., Hwang A.C., Lin M.H., Shimada H., Peng L.N., Loh C.H., Arai H., Chen L.K. (2018). Cognitive frailty and its association with all-cause mortality among community-dwelling older adults in Taiwan: Results from I-lan longitudinal aging study. Rejuv. Res..

[19] Makizako H., Shimada H., Park H., Doi T., Yoshida D., Uemura K., Tsutsumimoto K., Suzuki T. (2013). Evaluation of multidimensional neurocognitive function using a tablet personal computer: Test-retest reliability and validity in community-dwelling older adults. Geriatr. Gerontol. Int..

[20] Shimada H., Makizako H., Park H., Doi T., Lee S. (2017). Validity of the national center for geriatrics and gerontology-functional assessment tool and mini-mental state examination for detecting the incidence of dementia in older Japanese adults. Geriatr. Gerontol. Int..

[21] Shimada H., Tsutsumimoto K., Lee S., Doi T., Makizako H., Lee S., Harada K., Hotta R., Bae S., Nakakubo S. (2016). Driving continuity in cognitively impaired older drivers. Geriatr. Gerontol. Int..

[22] Folstein M.F., Folstein S.E., McHugh P.R. (1975). “Mini-mental state”. A practical method for grading the cognitive state of patients for the clinician. J. Psychiatr. Res..

[23] National Institute for Health and Care Excellence (2011). Donepezil, Galantamine, Rivastigmine and Memantine for the Treatment of Alzheimer's Disease, (TA217).

[24] Shimada H., Suzuki T., Suzukawa M., Makizako H., Doi T., Yoshida D., Tsutsumimoto K., Anan Y., Uemura K., Ito T. (2013). Performance-based assessments and demand for personal care in older Japanese people: A cross-sectional study. BMJ Open.

[25] Shinkai S., Watanabe S., Kumagai S., Fujiwara Y., Amano H., Yoshida H., Ishizaki T., Yukawa H., Suzuki T., Shibata H. (2000). Walking speed as a good predictor for the onset of functional dependence in a Japanese rural community population. Age Ageing.

[26] Cesari M., Kritchevsky S.B., Penninx B.W., Nicklas B.J., Simonsick E.M., Newman A.B., Tylavsky F.A., Brach J.S., Satterfield S., Bauer D.C. (2005). Prognostic value of usual gait speed in well-functioning older people—Results from the health, aging and body composition study. J. Am. Geriatr. Soc..

[27] Cesari M., Kritchevsky S.B., Newman A.B., Simonsick E.M., Harris T.B., Penninx B.W., Brach J.S., Tylavsky F.A., Satterfield S., Bauer D.C. (2009). Added value of physical performance measures in predicting adverse health-related events: Results from the health, aging and body composition study. J. Am. Geriatr. Soc..

[28] Studenski S., Perera S., Wallace D., Chandler J.M., Duncan P.W., Rooney E., Fox M., Guralnik J.M. (2003). Physical performance measures in the clinical setting. J. Am. Geriatr. Soc..

[29] Simonsick E.M., Newman A.B., Visser M., Goodpaster B., Kritchevsky S.B., Rubin S., Nevitt M.C., Harris T.B. (2008). Mobility limitation in self-described well-functioning older adults: Importance of endurance walk testing. J. Gerontol. Ser. A Biomed. Sci. Med. Sci..

[30] Fielding R.A., Vellas B., Evans W.J., Bhasin S., Morley J.E., Newman A.B., Abellan van Kan G., Andrieu S., Bauer J., Breuille D. (2011). Sarcopenia: An undiagnosed condition in older adults. Current consensus definition: Prevalence, etiology, and consequences. International working group on sarcopenia. J. Am. Med. Dir. Assoc..

[31] Morley J.E., Abbatecola A.M., Argiles J.M., Baracos V., Bauer J., Bhasin S., Cederholm T., Coats A.J., Cummings S.R., Evans W.J. (2011). Sarcopenia with limited mobility: An international consensus. J. Am. Med. Dir. Assoc..

[32] Chen L.K., Liu L.K., Woo J., Assantachai P., Auyeung T.W., Bahyah K.S., Chou M.Y., Chen L.Y., Hsu P.S., Krairit O. (2014). Sarcopenia in Asia: Consensus report of the Asian working group for sarcopenia. J. Am. Med. Dir. Assoc..

[33] Ministry of Health Labour and Welfare of Japan (2012). Annual Health, Labour, and Welfare Report 2011–2012.

[34] Verghese J., Lipton R.B., Katz M.J., Hall C.B., Derby C.A., Kuslansky G., Ambrose A.F., Sliwinski M., Buschke H. (2003). Leisure activities and the risk of dementia in the elderly. N. Engl. J. Med..

[35] Yesavage J.A. (1988). Geriatric depression scale. Psychopharmacol. Bull..

[36] De Jager C.A., Hogervorst E., Combrinck M., Budge M.M. (2003). Sensitivity and specificity of neuropsychological tests for mild cognitive impairment, vascular cognitive impairment and Alzheimer’s disease. Psychol. Med..

[37] Nestor P.J., Scheltens P., Hodges J.R. (2004). Advances in the early detection of Alzheimer’s disease. Nat. Med..

[38] DeCarli C., Mungas D., Harvey D., Reed B., Weiner M., Chui H., Jagust W. (2004). Memory impairment, but not cerebrovascular disease, predicts progression of MCI to dementia. Neurology.

[39] Hinrichs C., Singh V., Xu G., Johnson S.C. (2011). Predictive markers for ad in a multi-modality framework: An analysis of MCI progression in the ADNI population. NeuroImage.

[40] Gibbons L.E., Carle A.C., Mackin R.S., Harvey D., Mukherjee S., Insel P., Curtis S.M., Mungas D., Crane P.K., Alzheimer’s Disease Neuroimaging Initiative (2012). A composite score for executive functioning, validated in Alzheimer’s Disease Neuroimaging Initiative (ADNI) participants with baseline mild cognitive impairment. Brain Imaging Behav..

[41] Wild K., Howieson D., Webbe F., Seelye A., Kaye J. (2008). Status of computerized cognitive testing in aging: A systematic review. Alzheimers Dement..

[42] Verghese J., Wang C., Lipton R.B., Holtzer R. (2013). Motoric cognitive risk syndrome and the risk of dementia. J. Gerontol. Ser. A Biomed. Sci. Med. Sci..

[43] Wolf D.S., Gearing M., Snowdon D.A., Mori H., Markesbery W.R., Mirra S.S. (1999). Progression of regional neuropathology in Alzheimer disease and normal elderly: Findings from the nun study. Alzheimer Dis. Assoc. Disord..

[44] Buchman A.S., Schneider J.A., Leurgans S., Bennett D.A. (2008). Physical frailty in older persons is associated with Alzheimer disease pathology. Neurology.

[45] Newman A.B., Gottdiener J.S., McBurnie M.A., Hirsch C.H., Kop W.J., Tracy R., Walston J.D., Fried L.P. (2001). Associations of subclinical cardiovascular disease with frailty. J. Gerontol. Ser. A Biol. Sci. Med. Sci..

[46] Arvanitakis Z., Wilson R.S., Bienias J.L., Evans D.A., Bennett D.A. (2004). Diabetes mellitus and risk of Alzheimer disease and decline in cognitive function. Arch. Neurol..

[47] Puts M.T., Visser M., Twisk J.W., Deeg D.J., Lips P. (2005). Endocrine and inflammatory markers as predictors of frailty. Clin. Endocrinol..

[48] Weaver J.D., Huang M.H., Albert M., Harris T., Rowe J.W., Seeman T.E. (2002). Interleukin-6 and risk of cognitive decline: Macarthur studies of successful aging. Neurology.

[49] Ehl C., Kolsch H., Ptok U., Jessen F., Schmitz S., Frahnert C., Schlosser R., Rao M.L., Maier W., Heun R. (2003). Association of an interleukin-1beta gene polymorphism at position-511 with Alzheimer’s disease. Int. J. Mol. Med..

[50] Ma S.L., Tang N.L., Lam L.C., Chiu H.F. (2005). The association between promoter polymorphism of the interleukin-10 gene and Alzheimer’s disease. Neurobiol. Aging.

[51] Livingston G., Sommerlad A., Orgeta V., Costafreda S.G., Huntley J., Ames D., Ballard C., Banerjee S., Burns A., Cohen-Mansfield J. (2017). Dementia prevention, intervention, and care. Lancet.

[52] Ngandu T., Lehtisalo J., Solomon A., Levalahti E., Ahtiluoto S., Antikainen R., Backman L., Hanninen T., Jula A., Laatikainen T. (2015). A 2 year multidomain intervention of diet, exercise, cognitive training, and vascular risk monitoring versus control to prevent cognitive decline in at-risk elderly people (finger): A randomised controlled trial. Lancet.

[53] Gillette-Guyonnet S., Andrieu S., Dantoine T., Dartigues J.F., Touchon J., Vellas B., MAPT Study Group (2009). Commentary on “A roadmap for the prevention of dementia II. Leon Thal Symposium 2008.” The Multidomain Alzheimer Preventive Trial (MAPT): A new approach to the prevention of Alzheimer’s disease. Alzheimers Dement..

[54] Okura Y., Urban L.H., Mahoney D.W., Jacobsen S.J., Rodeheffer R.J. (2004). Agreement between self-report questionnaires and medical record data was substantial for diabetes, hypertension, myocardial infarction and stroke but not for heart failure. J. Clin. Epidemiol..

[55] Collard R.M., Boter H., Schoevers R.A., Oude Voshaar R.C. (2012). Prevalence of frailty in community-dwelling older persons: A systematic review. J. Am. Geriatr. Soc..

[56] Kojima G., Iliffe S., Taniguchi Y., Shimada H., Rakugi H., Walters K. (2017). Prevalence of frailty in Japan: A systematic review and meta-analysis. J. Epidemiol..

